# Langerin-expressing dendritic cells in pulmonary immune-related diseases

**DOI:** 10.3389/fmed.2022.909057

**Published:** 2022-09-07

**Authors:** Shurui Xuan, Yuebei Li, Yunhui Wu, Ian M. Adcock, Xiaoning Zeng, Xin Yao

**Affiliations:** ^1^Department of Respiratory and Critical Care Medicine, The First Affiliated Hospital of Nanjing Medical University, Nanjing, China; ^2^Airway Disease Section, National Heart and Lung Institute, Faculty of Medicine, Imperial College London, London, United Kingdom

**Keywords:** pulmonary immune-related disease, langerin, dendritic cells, immunity, pathogenesis

## Abstract

Dendritic cells (DCs) are “frontline” immune cells dedicated to antigen presentation. They serve as an important bridge connecting innate and adaptive immunity, and express various receptors for antigen capture. DCs are divided into various subclasses according to their differential expression of cell surface receptors and different subclasses of DCs exhibit specific immunological characteristics. Exploring the common features of each sub-category has became the focus of many studies. There are certain amounts of DCs expressing langerin in airways and peripheral lungs while the precise mechanism by which langerin^+^ DCs drive pulmonary disease is unclear. Langerin-expressing DCs can be further subdivided into numerous subtypes based on the co-expressed receptors, but here, we identify commonalities across these subtypes that point to the major role of langerin. Better understanding is required to clarify key disease pathways and determine potential new therapeutic approaches.

## Introduction

Langerin, a C-type lectin receptor (CLR) also known as CD207, was originally found to be highly expressed on Langerhans cells (LCs) that reside in the epidermis of the human skin (1). Later, the existence of langerin^+^ dermal DCs was clarified as well (2, 3). The disease with a relatively high association with langerin is Langerhans cell histiocytosis (LCH). LCH is a rare disease characterized by heterogeneous lesions, e.g., granulomatous lesions and histiocytosis X lesions, and the pathological features of affected tissues usually manifests as positive staining of CD1a and langerin (4, 5).

Langerin^+^ DCs were also found in tissues apart from skin including the lung, liver, kidney, and lymphoid tissue (6). To date, there is no comprehensive understanding of the specific function of these langerin^+^ DCs although LCs have some similar characteristics to other langerin-expressing DCs (7). However, these are not completely equivalent (7), e.g., murine epidermal LCs and dermal langerin-expressing DCs exhibit distinct repopulation kinetics and migratory characteristics *in vitro* and *in vivo*, and play distinct roles in humoral and cellular responses generated by gene gun immunization (7, 8). There are recent papers declaring that langerin-expressing DCs play a role in pulmonary immune-related disease settings. This article summarizes the immune and pathological relationship between langerin-expressing DCs and pulmonary immune-related diseases whose understanding will provide potential new therapeutic directions.

## The expression and characteristics of langerin in MNPs

Langerin was first recognized as an epitope specific to LCs by monoclonal antibody (mAb) DCGM4 staining (1, 9). Langerin mRNA is abundant in freshly isolated LCs while resting DCs generated from CD34^+^ progenitors treated with GM-CSF and TNFα are identified lower levels of langerin expression (1). LCs were traditionally regarded as a subset of immature DCs residing in epidermis and other mucosal epithelia due to their comparable function and dendritic processes (10, 11). Even though LCs are currently best classified as a type of mononuclear phagocytes (MNPs) distinct from DCs for that LCs are derived similarly to macrophages from the yolk sac during embryogenesis differently from DCs (12). According to the recent literature, we summarize the expression and distribution of langerin in the MNPs in tissue and blood of human and mouse. In human tissue, langerin is expressed by certain conventional DC2 (cDC2) cells from the dermis, lung, tonsil, and liver apart from epidermal LCs under healthy conditions, and it is rapidly induced in blood cDC2 upon tumor growth factor (TGF)-β stimulation (6, 13, 14). With the development of immunological knowledge of DC classification, human inflammatory blood DC3 were identified to express the langerin gene (15). It differs in murine normal tissue for that the expression of langerin was described in mouse cDC1 in view that the co-expressing CD103 (also named αE integrin) is a marker of cDC1 in mouse peripheral tissues (16, 17). Specifically, langerin was reported expressed by 15% of cDC1s in the murine lung (18).

The expression of langerin is regulated by various factors. Mononuclear cells can be induced to form LCs or LC-like DCs using factors such as GM-CSF and IL-4. Bone marrow-derived monocytes enter the peripheral blood and constitute 5% of circulating white blood cells. In response to appropriate stimuli, they migrate from the bloodstream into various peripheral tissues. A study has compared the responses to different maturation signals and antigen-presenting functions between LCs induced by GM-CSF and by M-CSF and demonstrated that GM-CSF can be replaced by M-CSF to some extent (19). Furthermore, TNF-α markedly increased the induction of langerin^+^ CD83^−^ LCs from both CD14-negative and CD14-positive precursors (20) whilst TGF-β1 can also affect the development of langerin^+^ epidermal LCs (21). In addition to some inflammatory factors that promote the increase of langerin, estrogen promotes the formation of a DC population with the unique features of epidermal LCs. The data suggest that differentiation of LCs *in vivo* will be dependent upon the local estrogen levels and estrogen receptor-mediated signaling events in the skin (22). Langerin^+^ cDCs and LCs are profoundly regulated by the retinoic acid (RA)-RA receptor (RARα) axis in a concentration-dependent manner (23). In addition to cytokines and growth factors such as GM-CSF and TGF-β1, the Notch receptor ligand Delta-1 is a regulator of the induction of human LC development from blood monocytes (24). Moreover, signaling by another Notch ligand JAG2 induces differentiation of CD14^+^ monocytes into LCH-like cells (25). In addition, inhibition of TNFAIP3, the negative regulator of NF-κB signaling affects Th cell differentiation in the presence of pulmonary langerin^+^ DCs (18).

Birbeck et al. elucidated the ultrastructure of LCs using electron microscopy (26). LCs have a lobular nucleus surrounded by a clear cytoplasm devoid of tonofilaments, desmosomes, or melanosomes. However, they possess an unique intracytoplasmic organelle which is their characteristic ultrastructural feature: the Birbeck granule (BG) (26, 27). Langerin is involved in the rapid internalization of BGs after mannose-binding *via* endocytosis. Intracellular tracing using an anti-langerin antibody demonstrated that following mannose-binding, langerin was internalized from the cell membrane into the BG (1). Indeed, the distribution and transport of langerin in immature LCs is mainly through the endosomal recycling of BGs. After internalization, langerin relocates back to the cell surface as part of a cell membrane-pericentriolar BG-cell membrane loop (28). Langerin appears to be a key structural element in BG formation due to langerin aggregation (1, 28), and presumably facilitates the uptake of mannans present on the cell surface of bacteria (9). BGs are characterized by the unusual cytoplasmic rod-like or tennis-racket-shaped structures, which can be visualized by electron microscopy as two apposed membranes separated by a striated zipper-like lamella (29). Langerin-deficient mice lack BG and the introduction of the langerin gene into embryonic fibroblasts induces the formation of BG (20). Oda et al. rebuilt the 3D structure of isolated BGs using cryo-electron tomography and identified a flexible loop region within langerin trimers that is crucial for BG formation and viral internalization (30).

Langerin polymorphisms affect both stability and sugar-binding activity. As such, langerin haplotypes may differ in their binding to pathogens and thus might be associated with susceptibility to infection. For example, the W264R form of langerin exhibits large changes in the structure of the CRD that alter its sugar-binding activity. In addition to structural factors, sugar-binding activity is also affected by other physical factors such as pH, temperature, and protein concentrations (31). Other mutations can result in thickened membrane structures compared with the typical cytomembrane sandwiching structures (CMS) of BG. In addition to BG structures, the affinity for high mannose glycoconjugates is to some extend affected (32).

## Immunological functions of langerin in MNPs

Advances in structural biology have provided evidence for the functional role of langerin. Langerin is a type II transmembrane cell surface receptor belonging to the Ca2^+^-dependent CLR family (33, 34). The extracellular domain (ECD) of langerin consists of a neck region containing a series of heptad repeats and a C-terminal C-type carbohydrate-recognition domain (CRD) featuring a glutamate-proline-asparagine (EPN) motif (position 285–287) (22, 35, 36). The extracellular region of langerin exists as a stable trimer kept together by a coiled coil of α-helices formed by the neck region. The CRD exhibits selectivity for mannose, N-Acetylglucosamine (GlcNAc), and fucose (37), but only the trimeric ECD fragment binds to glycoprotein ligands. The ECD binds human high-mannose oligosaccharides as well as yeast invertase mannose-containing structures but not complex glycan structures (36). After antigen capture, langerin internalizes the antigen, e.g., Candida albicans (38), Mycobacterium leprae (39), or HIV-1 (40) *via* the BG. Considering that the expression levels of langerin was markedly reduced along with LCs maturation, Valladeau et al. further confirmed that Langerin is restricted to immature DC (41). While Stoitzner et al. demonstrated that certain expression of langerin on the surface of matured and emigrated DCs were retained in a time-course-dependent manner which suggested that LCs or other langerin^+^ DCs can be traced to the draining lymph nodes by their langerin expression (42). In recent research, langerin were used as one of the markers for immature monocyte-derived DCs (moDCs) (43).

As a sugar-binding protein expressed on the surface of DCs, langerin has a key role in antigen-uptaking when DCs serve as professional antigen-presenting cells to exert immune function. In leprosy, for example, LC-like DCs and freshly isolated epidermal LCs present non-peptide antigens of Mycobacterium leprae to T cell clones derived from a leprosy patient in a CD1a-restricted and langerin-dependent manner (39). In addition, GM-CSF-dependent langerin^+^ CD103^+^ dermal DCs promote CD4^+^ effector Th cell differentiation and play a role in autoimmune pathogenesis (44). Human primary LCs capture the measles virus (MV) through langerin, which then presents MV-derived antigens in the context of HLA class II to MV-specific CD4^+^ T cells independent of CD8^+^ T cells (45). However, the evidence for a critical role of langerin^+^ DCs in CD8^+^ T cell activation do exist after gene gun DNA vaccination as well (46). LCs and cDC1s can mediate different humoral immune response through Langerin which may give us inspiration in development of vaccine effectiveness (47).

Langerin has been proposed as a frontline sentinel in the immunization process, e.g., HIV transmission (48) and Inflammatory Bowel Disease (49). As such, LC-DC clustering *via* langerin leads to DC maturation and facilitates antigen transfer of HIV-1 to DCs, which subsequently induces activation of CD8^+^ T cells (50). In contrast, it has been proposed that HIV-1 captured by langerin is internalized into BGs and then degraded. This would suggest that langerin does not enhance HIV-1 infection of T cells but rather prevents T-cell infection by viral clearance (51). Yet some research has conclusively shown that HIV was effectively transmitted to the primary target CD4^+^ T cells (52–54). The demonstration was confirmed in subsequent studies by Bertram groups (55, 56). Furthermore, langerin was revealed to induce HIV-1 specific humoral immunity in addition to cellular immunity (57). Further research is required in the area to define whether langerin promotes or inhibits immunity and under which specific conditions and if these specific conditions can be artificially controlled. This may open up new avenues for clinical prevention and treatment.

## Langerin-expressing DCs in pulmonary immune-related diseases

Within the human lung, langerin is mainly expressed on the lung mucosa and the vascular wall (16). More specifically, staining is seen within the airway epithelium, lung parenchyma, visceral pleura (58), and lung draining LN (DLN) (6), and similar results are obtained in the mouse (33). Despite the link between skin and lung disease through the atopic march (59), research into the function of langerin in the lung and airway immune-related disease has not been studied in depth with much relating to the analysis of relative expression profiles in disease. Nevertheless, we can still speculate on the possible role of langerin based on the available evidence as shown in Table 1 to inspire more further research.

**Table 1:** Potential functions of langerin in pulmonary diseases.

**Pulmonary diseases**	**Langerin potential functions**	**Immune trend**
Lung carcinoma	Tumor peptides cross-presentation	CD8^+^ T cell activation (61)
Asthma	Induce and maintain Th2 response	CD4^+^ T cell activation (68)
Pulmonary fibrosis and PLCH	Pathogenic mutations	MAPK signaling alterations (86)
COPD	Induce Th1 response reacting to CS	CD4^+^ T cell activation (89)
Microbial infection	Pathogen scouting	Neutrophil and macrophage recruitment (100)

CS, Cigarette smoking; MAPK, Mitogen-activated protein kinase.

### Lung carcinoma

Early studies examined langerin expression in bronchial biopsies of primary lung carcinomas from 12 patients and found infiltration of DCs within tumor tissues including LCs and CD1a^+^/langerin^+^ cells interspersed among tumor cells (60). More recent research using high-throughput sequencing has provided a more complete picture of langerin expression in lung cancer. The depletion of langerin^+^ DCs before and after vaccination with VLP-gp33r (a lymphocytic choriomeningitis virus–derived peptide antigen) inhibits the growth of Lewis' lung carcinoma tumors expressing gp33 (LL-LCMV), leading to reduced cytotoxic CD8^+^ T cell activity. This highlights the importance of langerin in antigen cross-presentation of tumor peptides (61). In general, langerin plays a positive role in promoting the immune response during tumor immunity, which has been seen in other tumors such as oral cavity primary squamous cell carcinoma (62). In breast cancer tissues, CD1a and langerin staining was found in one-third of primary tumors but this did not correlate with clinicopathological data (63). This was divergent from previous findings from the same group that most LCs were resident within all tumor samples (64). However, with the update of cognition of immunological markers, CD1a and langerin expression may not discriminate LCs from langerin^+^ cDC2 or CD11c^+^ epidermal DCs (48, 55). It may lead to completely different conclusions in subsequent research.

In addition, tumor cells were found to promote langerin expression. Some, but not all, lung carcinomas produced GM-CSF and a good correlation exists between GM-CSF production and the number of CD1a^+^ LCs infiltrating these tumors (65). Breast cancer cells can chemoattract CD34^+^ progenitor cells through CCL20/MIP3α and promote the differentiation of progenitor cells into langerin^+^ DCs depending upon the level of TGF-β present. These langerin^+^ DCs differentiate into two types: CD1a^+^ langerin^+^ CD86^+^ and CD1a^high^ langerin^−^ CD86^−^ cells (66).

**Figure 1:**
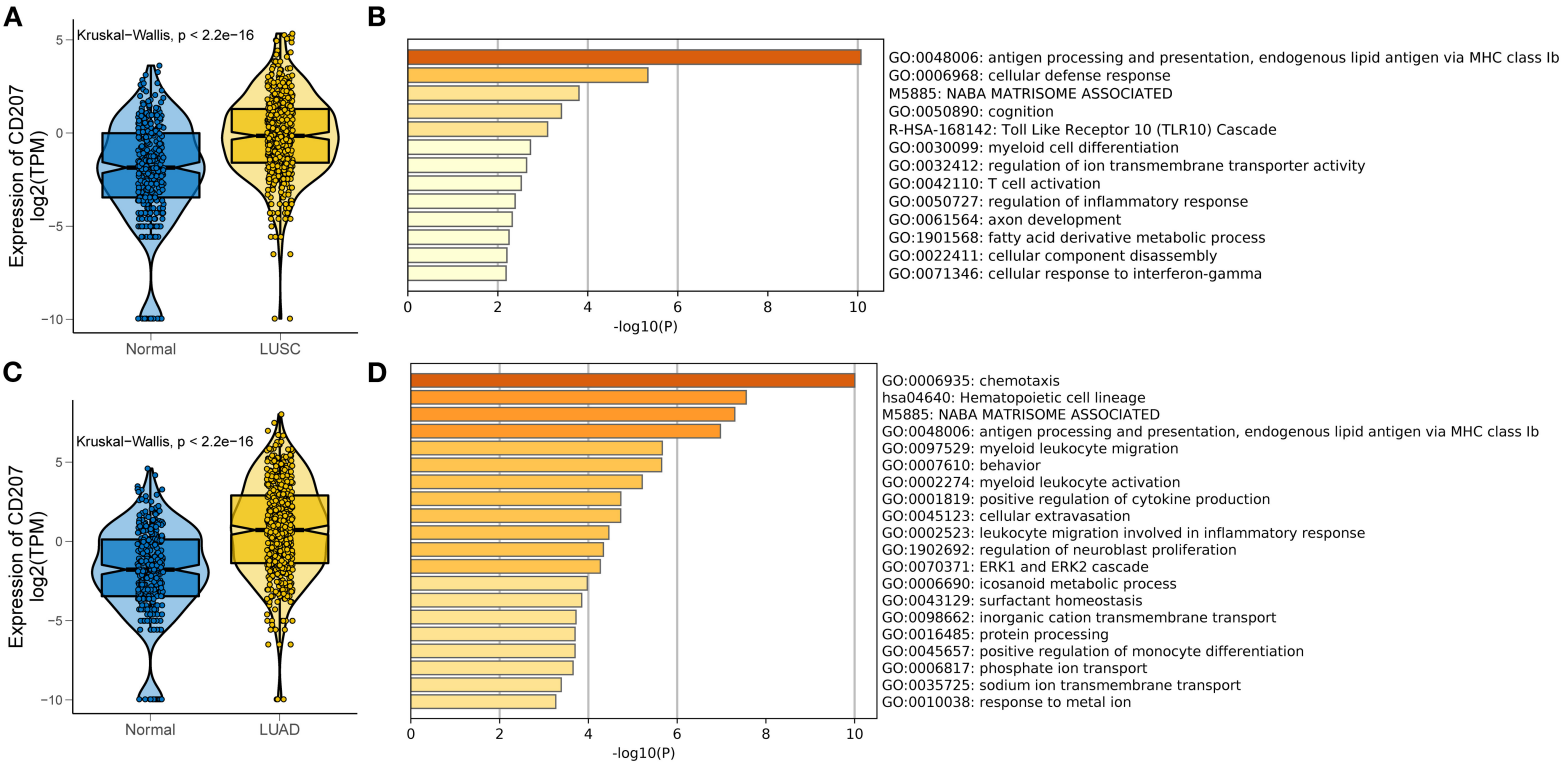
Expression and functional annotation of langerin in LUSC and LUAD in the TCGA RNA-seq datasets. **(A,C)** Boxplots visualizing significantly different expressions of langerin between normal and LUSC (and LUAD) tissues in TCGA database. **(B,D)** Bar plot showing the top 20 terms derived from the gene set enrichment of the differentially expressed genes in TCGA-LUSC (and LUAD) samples. The x-axis represents statistical significance.

Using The Cancer Genome Atlas (TCGA) we analyzed the expression of langerin in Lung Squamous Cell Carcinoma (LUSC) (Figure 1A) and Lung Adenocarcinoma (LUAD) (Figure 1C) to identify any differential expression and the pathways associated with langerin up-regulation. Using this large database, we found significant up-regulation of langerin in both LUSC and LUAD which was associated with distinct gene ontology (GO) molecular pathways only some of which overlapped. The analysis showed that langerin in the two types of lung tumor was both positively correlated with immunity related signaling pathways, e.g., antigen processing and presentation, and endogenous lipid antigen *via* MHC class Ib (Figures 1B,D). These data provide a basis for further research on the role of langerin in tumor pathogenesis.

### Asthma

In asthma, DCs are important not only for inducing T helper (Th) 2-cell sensitization but also for maintaining effector Th2-cell responses during ongoing allergic disease (67). In a mouse model of house dust mite-induced allergic asthma, subsequent LPS exposure resulted in enhanced migration of langerin^+^ DCs from the lung to the draining lymph node and LPS-exposed langerin^+^ DCs instructed CD4^+^ T cells toward a Th2 response. Selective depletion of langerin^+^ DCs prevented LPS-induced eosinophil recruitment and T-cell activation (68). In addition, langerin expression was up-regulated in induced sputum from asthmatic subjects and correlated with airway coagulation factor XIII (FXIII) and airflow limitation (69). Moreover, asthmatic human respiratory-tract DCs (hRTDC) expressed significantly higher levels of langerin than equivalent cells from control subjects. In addition, langerin^+^ cells from sputum co-cultured with naive T cells increased T cell proliferation 2.5-fold (70). These results suggest potential langerin-specific novel therapeutic approaches for the treatment of severe asthma with irreversible airflow obstruction.

The largest lung DC population are the integrin αEβ7 positive and I-A^high^ CD11c^high^-DC population which express high levels of langerin and act to enable efficient antigen uptake and presentation (16). However, in an analysis of large airways and bronchopulmonary LNs in fatal asthma (FA), there were no statistical differences in the expression of langerin^+^ DCs between the FA patients and control subjects (71). These differences in the expression of langerin^+^ DCs in different studies may be due to analysis of different asthma immunophenotypes and/ or an effect of therapy. In addition, different sampling locations such as sputum, BALF, large airways, and bronchopulmonary LNs may contain different numbers of langerin^+^ DCs. Therefore, further research is needed to elucidate the role of langerin in the pathogenesis and progression of asthma taking disease severity and subphenotypes into account.

### Pulmonary fibrosis and pulmonary LCH

Pulmonary fibrosis is an umbrella term that covers idiopathic pulmonary fibrosis (IPF) and non-specific interstitial pneumonia, importantly the characteristics of the immature DCs that infiltrate during fibrosis and epithelial hyperplasia in these diseases are similar (72). Intraepithelial infiltrating CD1a^+^/langerin^+^ DCs committed to mucosal immunologic surveillance (73). High levels of langerin staining are also seen in sites of fibrosis in PLCH, which may indicate an important connection between langerin expression and the pathological changes of fibrosis (74).

In adults, pulmonary LCH (PLCH) occurs predominantly in young smokers or ex-smokers (>90% of cases) with a peak incidence between the ages of 20 and 40 (75, 76). Patients with PLCH develop shortness of breath, pleuritic pain, or spontaneous pneumothoraces. There is evidence of obstruction, air trapping, and decreased carbon monoxide diffusing capacity (DLCO) which may help to identify patients with a poor prognosis (77). High-Resolution Computed Tomographic (HRCT) imaging of the chest is critical in the diagnosis of suspected PLCH and typically shows a combination of nodules, cavitated nodules, thick- and thin-walled cysts (78–80). Transbronchial lung biopsy is diagnostic in about 30% of cases and is valuable in excluding other diagnoses that mimic PLCH (81). PLCH presents as accumulation of LCs and other langerin-expressing DCs in the lungs (82). A comparison of BAL samples of patients with PLCH, sarcoidosis or IPF, found that patients suffering from PLCH had a significantly higher number of CD1a^+^ and langerin^+^ cells than the subjects with sarcoidosis and IPF (74).

A similar comparison was made between LCH and other interstitial, inflammatory, and infectious diseases as well. Counting the number of cells staining per high power field (400 x) in areas of highest density indicates that the number of CD1a^+^ and langerin^+^ cells in LCH lesions is more than two-fold that in interstitial pneumonia (83). Additional research has reported that Langerhans-like CD1a^+^ cells are present with NRAS and BRAF mutations in patients (84), providing new insights into the pathogenesis of the disease. To date, langerin has been used as a diagnostic index of PLCH (85), but its true role in the pathophysiology or the intrapulmonary mechanism of the disease requires elucidation. A recently published report presents consensus recommendations that resulted from the discussions at the annual Histiocyte Society meeting in 2019 (5) which propose that the single-system PLCH may indeed be a clonal process for that recurrent Mitogen-activated protein kinase (MAPK) pathway alterations and BRAF-V600E mutations have been identified in lesions (86, 87). All questions are urgent for answers.

### Chronic obstructive pulmonary disease

There is a significantly higher expression of langerin mRNA in human COPD lung tissue compared with those from healthy control subjects (88). The primary cause of COPD for most subjects is tobacco smoking, with other causes being air pollution and genetics. Interestingly, immunohistochemical staining of langerin expression in the small airways revealed more LC-type DCs (identified by langerin and the presence of BG) in current smokers without COPD and in COPD patients, vs. never smokers and ex-smokers without COPD (89). PLCH, which occurs predominantly in young smokers or ex-smokers, has high langerin positivity as a diagnostic indicator and shows the pathological manifestations in end-stage disease (dense fibrosis, cystic changes, and honeycomb lungs) that are similar to that of emphysema. This suggests that langerin is involved in the pathophysiology of COPD particularly with lung destruction and airway remodeling. Taniguchi et al. firstly generate a glycosyltransferase, α1,6-fucosyltransferase (Fut8) knockout mice to discover COPD-like phenotypes in mouse model (90–93). To develop an effective clinical therapy application, candidate glycan keratan sulfate (KS) and the di-sulfated KS disaccharide L4, which were identified as specific glycan ligands to Langerin (94). It was supposed that KS-based glycomimetics may protect hijacking by viruses or bacteria in a langerin-dependent manner (95).

### Microbial infection

Mediastinal lymph nodes contain increased numbers of cells co-expressing langerin and CD103 when the lung is infected with the virus, and depletion of lung langerin^+^ DCs in langerin-DTR mice aggravates the severity of infection (96). Study of viral infection reveals that CD103^+^ langerin^+^ double-positive dermal DCs and langerin^+^ epidermal LCs firstly upregulate innate immune response in the draining lymph node (97). CD103 binds integrin β7-ITGB7 to form the complete heterodimeric integrin molecule αEβ7 that the chief ligand is epithelial cellular adhesion molecule E-cadherin. Some CD103-expressing immune cells primarily reside on the epithelium in order to rapidly respond to both viral and bacterial infection (98). The considerable co-expression of langerin and CD103 inspire that whether there is related regulation of their, respectively, expression and whether αEβ7/E-cadherin-interaction enhance the receptor function of langerin.

As mentioned above, langerin is able to capture virus particles including HIV although the precise mechanism involved is unclear. Studies are underway in procaine models of infection to elucidate the key pathways by which langerin impacts lung viral infection (99). Furthermore, there is evidence that langerin plays a role in pathogen sensing, neutrophil and macrophage recruitment, and the downstream inflammatory processes. This is an exciting area for future research that may provide novel non-macrolide work therapeutic targets for acute exacerbations of lung diseases (100).

## Conclusion

The current understanding of the role of langerin-expressing DCs in pulmonary diseases is lacking details although the evidence suggests that langerin plays a role in both the immuno-inflammatory aspects of the disease as well as on structural remodeling and exacerbations. DCs are key cells in initiating adaptive immunity and langerin acts as its surface receptor to sense external stimuli. However, it is clear that langerin possesses additional functions that make it an interesting target for future research. Considering the important role played by DCs in the pathogenesis of immune disorder of the lungs and airways, a deeper insight into langerin mechanisms may provide novel therapeutic modalities for immune and structural aspects of pulmonary immune-related diseases.

## Author contributions

Conception and design: XY, SX, and YW. Manuscript writing: SX. Data analysis and interpretation: YL. Collection and assembly of literature: SX and YW. Language editing and proofreading: IA and XZ. Final approval of manuscript: all authors.

## Funding

This research was supported by National Natural Science Foundation of China (No. 81870039).

## Conflict of interest

The authors declare that the research was conducted in the absence of any commercial or financial relationships that could be construed as a potential conflict of interest.

## Publisher's note

All claims expressed in this article are solely those of the authors and do not necessarily represent those of their affiliated organizations, or those of the publisher, the editors and the reviewers. Any product that may be evaluated in this article, or claim that may be made by its manufacturer, is not guaranteed or endorsed by the publisher.
